# Association between diabetes and cognitive function at baseline in the Brazilian Longitudinal Study of Adult Health (ELSA- Brasil)

**DOI:** 10.1038/s41598-020-58332-9

**Published:** 2020-01-31

**Authors:** Mônica M. Teixeira, Valéria M. A. Passos, Sandhi M. Barreto, Maria I. Schmidt, Bruce B. Duncan, Alline M. R. Beleigoli, Maria J. M. Fonseca, Pedro G. Vidigal, Larissa F. Araújo, Maria de Fátima H. S. Diniz

**Affiliations:** 1 Faculty of Medicine, Universidade Federal de Minas Gerais, Belo Horizonte, Brazil; 2 Faculty of Medicine, Universidade Federal do Rio Grande do Sul, Porto Alegre, Brazil; 3 Public Health School, Fundação Oswaldo Cruz, Rio de Janeiro, Brazil; 4 Faculty of Medicine, Faculdade de Ciências Médicas de Minas Gerais, Belo Horizonte, Brazil; 5 Public Health School, Universidade Federal do Ceará, Fortaleza, Brazil; 6 Flinders University, Adelaide, Australia

**Keywords:** Cognitive ageing, Diabetes complications

## Abstract

Diabetes has been associated with cognitive changes and an increased risk of vascular dementia and Alzheimer’s disease, but it is unclear whether there are associations between diabetes and early alterations in cognitive performance. The present study consisted of a cross-section analysis of 14,444 participants aged 35–74 years and from a developing country at baseline in the Brazilian Longitudinal Study of Adult Health (ELSA–Brasil); these participants were recruited between 2008 and 2010. We investigated whether there was an association between diabetes and early changes in the cognitive performance of this Brazilian population. To assess cognitive domains, we used the word-list learning, word-list delayed recall and word recognition tests along. Phonemic verbal fluency tests included semantic phonemic test (animals) and a phonemic test (words beginning with the letter F). Executive functions associated with attention, concentration and psychomotor speed were evaluated using the Trail Making Test B. The exposure variable in the study was defined as diabetes. Multiple linear regression was used to estimate the association between diabetes and cognitive performance. The results were adjusted for age, sex, education, hypertension, coronary disease, depression, physical activity, smoking, alcohol consumption, and the cholesterol/HDL-C ratio. We found a significant association between diabetes and decreased memory, language and executive function (attention, concentration and psychomotor speed) performance in this population from a country with a distinct epidemiological profile, even after adjusting for the main intervening variables.

## Introduction

The world population is ageing, and the incidence of neurodegenerative diseases associated with age is increasing. Diabetes mellitus (diabetes) has been associated with cognitive changes and an increased risk of vascular dementia^1^ and Alzheimer’s disease^2^. The number of people diagnosed with diabetes worldwide is approximately 422 million individuals^3^. Cognitive dysfunction in individuals with diabetes can result from interactions between inherent metabolic abnormalities, such as hyperglycaemia, hyperinsulinaemia, and micro- and macrovascular complications, in addition to hypertension, dyslipidaemia, depression and obesity^4–7^. The precise mechanisms involved in degenerative diseases in patients with diabetes are unknown and not fully understood; thus, they are considered quite complex and dynamic^8^.

Some studies show a worse performance in cognitive tests among patients with diabetes than among individuals without diabetes, with deficits in several domains, especially executive function, memory, psychomotor speed and attention^9,10^. A systematic review that included case-control and population-based studies showed that the risk of overall cognitive dysfunction was increased in people with diabetes in five of seven cohorts. In addition, the association of decreased cognitive performance in one or more domains in was reported in 13 of the 20 cross-sectional studies and in five of the seven longitudinal studies included in this review^11^. According to Berg *et al*., the association between diabetes and cognition differed among the domains; the processing speed was significantly affected in 63% of the studies; attention, in 50%; memory, in 44%; cognitive flexibility, in 38%; one language, 33% and general intelligence in 31%^11^. These functions are particularly relevant because they involve behaviours such as problem solving, judgement, and changing habits. All these functions are important in prescribing complex tasks, such as aligning the insulin dose with the carbohydrate content, predicting the impact of physical activity on blood glucose, or even recognizing and treating hypoglycaemia and hyperglycaemia appropriately^12,13^.

Many studies have investigated the association of diabetes and cognition only in elderly populations^2,5,7^. Recognizing early changes in cognition tests in patients with diabetes may be important for potential future interventions that help mitigate unfavourable consequences, thereby improving the management of these problems.

This study has the purpose of assessing whether there is an association between cognitive test performance and diabetes at baseline in a large cohort of young and middle-aged individuals in a developing country that has been experiencing a sociodemographic and nutritional transition in the last three decades. The relevance of this study deserves to be highlighted; this is a Latin American cohort, and there are increasing incidence rates of diabetes and cognitive impairment in middle and low-income countries^14^.

## Material and Methods

### Study population and ethics

The present study is a subproject of the Longitudinal Study of Adult Health (ELSA–Brasil). The baseline data was collected from 2008 to 2010 in public universities and research institutions in six Brazilian capitals: the Universidade Federal da Bahia (UFBA), Universidade Federal de Minas Gerais (UFMG), Centro Federal de Educação Tecnológica de Minas Gerais (CEFET-MG), Universidade Federal do Espírito Santo (UFES), Fundação Oswaldo Cruz (FIOCRUZ), Universidade de São Paulo (USP) and Universidade Federal do Rio Grande do Sul (UFRGS). The purpose of the ELSA-Brasil study was to investigate the risk factors and incidence of chronic diseases, especially diabetes and cardiovascular diseases^15^. The data collection was performed via face-to-face interviews, anthropometric measures, arterial pressure and laboratorial tests in the six research centres^16^.

**Figure 1:**
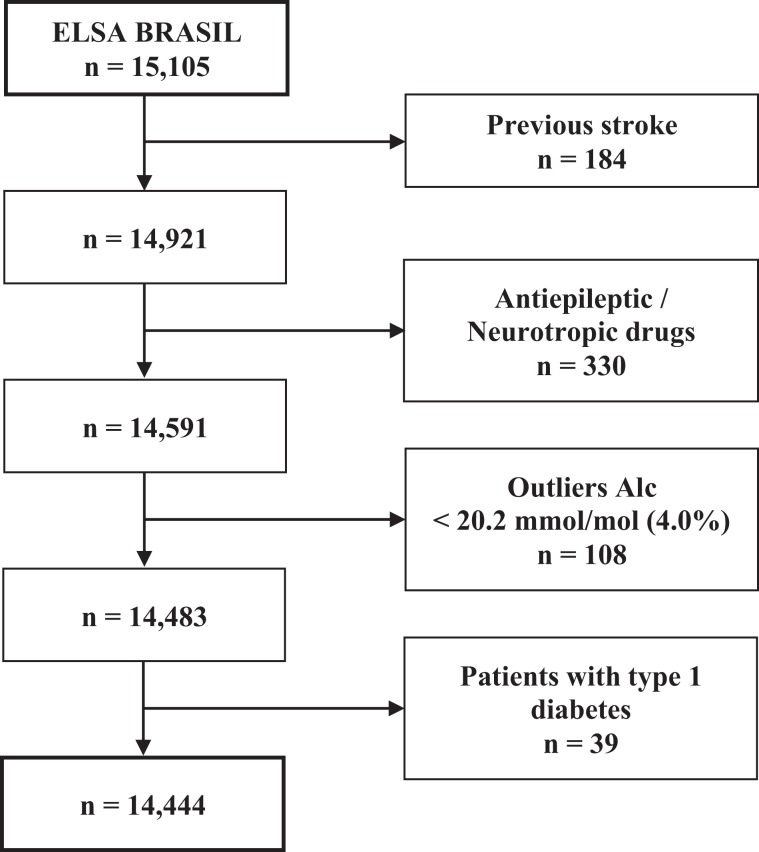
The process of selection of the study population.

The baseline ELSA-Brasil cohort included 15,105 individuals aged between 35 and 74 years; 54.3% were women, 17.3% had diabetes, 35.4% had hypertension, 22.9% were obese, and 40.2% were overweight. In the present analysis, 184 participants were excluded due to a previous history of stroke; 330 were excluded due to their use of antiepileptic medications, neurotropic agents, or psychotropic agents that could interfere in the cognitive tests; and 108 were excluded due to a glycated haemoglobin (A1c) level below 4.0%. We excluded 39 patients with type 1 diabetes. The remaining final sample size was 14,444 individuals (Fig. 1).

All participants signed the free informed consent form from the ELSA-Brasil study. This study was approved by the Ethics and Research Committees of all institutions, including the Research Ethics Committee of the Federal University of Minas Gerais, Belo Horizonte (ETIC 186/06), and the National Research Ethics Committee. This study was approved by the National Commission of Ethics in Research (CONEP), and all methods were conducted according to the relevant guidelines and regulations of this cohort^16^.

### Tests of cognitive function (response variables)

Cognitive performance was assessed using internationally standardised tests. The Brazilian version of the learning, recall and word recognition test of the Consortium to Establish a Registry for Alzheimer’s Disease (CERAD) includes a list of ten unconnected words that was showed to the participant. Immediately afterwards, the participant had to recall as many words as possible. This procedure was performed three times to evaluate immediate recall (word-list learning). This test evaluates the retention of learned words by delayed recall after approximately five minutes, during a second phase of the test. Word recognition was assessed by a list of 20 words (ten from the initial stage mixed with ten other words). Participants had to recognise the greatest possible number of words from the initial phase (word recognition test). Memory is a sum of learning, recall and word recognition tests^17^.

Verbal fluency was assessed by tests that evaluate language, memory and executive functions. Verbal fluency tests include a semantic phonemic test (animals) and a phonemic test (words beginning with the letter F). These tests ask the participant to say all the words related to the category (animals) and beginning with the letter F that they can remember within one minute, with final scoring following standardization rules^17^.

Executive functions associated with attention, concentration and psychomotor speed were evaluated using the Trail Making Test B, which requires the participant to draw a line connecting numbers and words as quickly as possible without lifting the pencil from the paper. The task execution time is then noted^17^. Version A of Trail Making Test B was used as training for version B (Trail B), as pre-tests revealed that many participants had difficulty understanding this task^17–21^.

### Diabetes (explanatory variable)

Samples were collected after a 12-hour fast, stored in a freezer at −80 °C and sent to the certified central laboratory in São Paulo. An oral glucose tolerance test (OGTT) was administered to all participants without a known diabetes diagnosis [15]. Glycaemia was measured using the enzymatic colorimetric method (ADVIA 1200 Siemens Deerfield, IL, USA), and glycated haemoglobin A1c was measured using *high-pressure chromatography* (HPLC - Bio-Rad Laboratories, Hercules, CA, USA).

Diabetes was defined as A1c ≥ 6.5% (48 mmol/mol), fasting glycaemia ≥ 126 mg/dL (7.0 mmol/L), or OGTT ≥ 200 mg/dL (11.1 mmol/L), according to the American Diabetes Association (ADA) criteria; by insulin and antidiabetic drug use; or by the self-reported medical diagnosis of diabetes.

### Covariates

We investigated and categorised sociodemographic variables such as age, sex and education. Age was grouped as follows: 35 to 44, 45 to 54, 55 to 64 and ≥65 to 75 years old. Education was divided into four sub-groups: <8 years (incomplete fundamental school), eight years (complete fundamental school), 9–11 years (high school) and ≥12 years (university degree)^22^. Leisure time physical activity was assessed using the long modified version of the International Physical Activity Questionnaire (IPAQ) and was categorised as low, moderate and high^23^. Smoking *status* was categorised as never, former, and current smoker. Alcohol use was categorised as low (weekly consumption ≤175 g), moderate (176–350 g weekly consumption) and high (>350 g weekly consumption).

To assess adiposity, we used anthropometric data and applied standardised techniques and equipment. Weight (kg) and height (m) were measured with the participant barefoot and wearing light clothes using a Toledo™ scale (accuracy 100 g) and a stadiometer (accuracy 0.1 cm), respectively. Body mass index was also determined [weight (kg)/height (m^2^)]. Abdominal circumference (midpoint between the last rib and the iliac crest) was measured using an inelastic tape (cm), and the average of two measures was used in the analysis. The measures were confirmed by trained and certified study personnel according to the standards of the project^24^.

Arterial hypertension was defined by a self-reported medical diagnosis of hypertension, use of anti-hypertensive drugs, or blood pressure ≥140/90 mmHg at the time of evaluation (sum of two measures). Coronary heart disease was defined as a history of myocardial revascularization and/or myocardial infarction. Depression was defined by the use of antidepressant drugs at the time of the interview.

Total cholesterol was determined by the enzymatic colorimetric method; high-density cholesterol (HDL-C) was determined by the homogeneous colorimetric method without precipitation, and triglycerides were determined by glycerol-phosphate peroxidase (ADVIA 1200 Siemens Deerfield, IL, USA). We used the total cholesterol/HDL-C ratio as a covariate^25^.

### Statistical analysis

The statistical analysis was accomplished using STATA™ software (Stata Corporation, College Station, TX, USA), v. 14.0. The categorical variables were described as frequencies and percentages. The continuous variables were described by the means and standard deviations (SDs) or medians and interquartile ranges, according to the normality of data definition, which was tested in histograms, and coefficients of asymmetry (skewness) and kurtosis. In the univariate analysis, Pearson and Spearman’s correlations were used for the continuous variables with normal and non-normal distributions, respectively, to assess the correlation between the potential factors and cognitive performance. To compare means and medians from the cognitive tests among the potential associated factors, we used Student’s t-test, the Mann-Whitney test, ANOVA or the Kruskal-Wallis test. All the variables with *p* < 0.20 in the univariate analysis were pre-selected for inclusion in the multivariate analysis. The Trail Making Test B had a skewed distribution; therefore, we used the natural logarithmic transformation (log-transformed).

Multiple linear regression (forward) was applied for response variables memory test, verbal fluency test (animals and letter F), and log-transformed Trail Making Test B to estimate the association between cognitive performance and diabetes (yes/no). The data were adjusted for age (categorical), sex and education in model 1; for all model 1 variables plus hypertension, coronary disease, and depression in model 2; and for all model 2 variables plus physical activity, smoking, alcohol use, and total cholesterol/HDL-C ratio in model 3.

## Results

There were 14,444 participants in this study; 54% were female, and the mean age was 52.0 years (±9.1). The education level was high; 52.8% of the participants had a university degree. The characteristics of the participants are depicted in Table 1: 76.7% were sedentary, 57.0% never smoked, 63.1% were overweight (BMI 25.0–29.9 kg/m^2^), and 22.8% were obese (BMI ≥ 30.0 kg/m^2^). In the univariate analysis, there was an association between a worse cognitive performance in all cognitive tests and the highest age group, decreased schooling and smoking. The variables with p < 0.2 in the univariate analysis were maintained in the multivariate analyses: hypertension, physical activity, alcohol use, coronary disease, and the total cholesterol/HDL-C ratio.

**Table 1:** Characteristics of the participants of the study- ELSA-Brasil (2008–2010). *Note*. Memory = word-list learning + word-list delayed recall + word recognition tests. Data are presented as mean ± SD, median [IQ] or n (%). N: number; %: percentage; SD: standard deviation; IQ: interquartile range. A1c: glycated hemoglobin; Total Col-HDL-C – total cholesterol/High density cholesterol ratio.

Variables	Total population N = 14,444
Demographics	
Female n (%)	7,850(54.4)
Age (years), n (%)	
35 a 44	3,233(22.4)
45 a 54	5,687(39.4)
55 a 64	4,036(28.0)
65 a 74	1,488(10.2)
Education, n (%)	
Incomplete fundamental	840(5.8)
Complete fundamental	982(6.8)
High school	4,991(35.0)
University degree	7,631(52.8)
Cognitive function	
Word-list learning mean ± SD	21.1 ± 3.9
Word-list delayed recall median[IQ]	7 [6–8]
Word recognition test median[IQ]	10[9–10]
Memory (learning, recall and word recognition test)	37.7 ± 5.9
Phonemic verbal fluency test (letter F) mean ± SD	12.5 ± 4.5
Semantic phonemic test (animals) mean ± SD	18.5 ± 5.3
Trail-making test B median[IQ]	95[73–132]
Habits and comorbidities	
Physical activity level, n (%)	
Low	10,913(76.7)
Moderate	2,317(16.3)
High	1,006(7.1)
Smoking, n (%)	
Never	8,234(57.1)
Former	4,335(30.0)
Current	1,874(13.0)
Alcohol consumption (g/week), n (%)	
≤175	13,119(90.9)
176–350	939(6.5)
>350	383(2.7)
Diabetes n (%)	
No	11,999(83.1)
Yes	2,442 (16.9)
Hypertension, n (%)	
No	9,331(64.6)
Yes	5,099(35.4)
Coronary artery disease, n (%)	
No	13,399(96.8)
Yes	439(3.2)
Depression, n (%)	
No	13,649(95.0)
Yes	795(5.0)
Markers of adiposity, and laboratorial tests	
Body mass index,(kg/m^2^)	27.0 ± 4.7
Waist circumference (cm)	91.2 ± 12.8
A1c mmol/mol(%)	36.6 ± 12.0 (5.5 ± 0.96)
Fasting plasma glucose (mg/dl)	106.7 ± 29.2
Triglycerides (mg/dL) median[IQ]	114.0[81–166]
HDL-C (mg/dl) mean ± SD	56.7 ± 14.5
Total Col-HDL-C median[IQ]	3.9[3.2–4.6]

Table 2 shows the result of the linear regression between the memory tests (learning, recall and word recognition tests) and diabetes. In the final model, we found that a lower cognitive performance was associated with diabetes. The results also showed better cognitive performance in women, and poor cognitive performance was associated with increased age, decreased schooling, smoking, moderate consumption of alcohol, and increased total cholesterol/HDL-C ratio. In Tables 3 and 4, we present the results of the linear regression analysis between verbal phonemic fluency (letter F), semantic phonemic test (animals) and diabetes. In this executive domain, the final model shows a worse cognitive performance with diabetes. Decreased cognitive performance was associated with increased age, among men, with decreased education, hypertension, depression, smoking and decreased physical activity levels.

**Table 2:** Multivariate regression final models with memory function test and diabetes in ELSA-Brasil (baseline 2008–2010) n = 14,444. *Note*. Total Col-HDL-C-Total cholesterol/High density cholesterol ratio. β[CI95%] – coefficient β and 95% confidence interval; R^2^-R^2^ adjusted. Model 1- Adjustment by age, sex and education, Model 2 - Model 1 plus hypertension, coronary disease and depression. Model 3 - Model 2 plus physical activity, smoking, alcohol use, total cholesterol / HDL-C. *p < 0.05; **p < 0.01; ***p < 0.001 Final models included variables with p < 0.05.

Variable	Memory test (learning, recall and word recognition test)			
	Model 1 β[CI 95%] R^2^0.21	Model 2 β[CI 95%] R^2^0.21	Model 3 β[CI 95%] R^2^0.22	Final Model β[CI 95%] R^2^0.21
Diabetes	−0.58 [−0.82, −0.34]***	−0.58 [−0.82, − 0.34]***	−0.57 [−0.82, − 0.31]***	−0.53 [−0.77, − 0.29]***
Age (years)				
35 a 44	1	1	1	1
45 a 54	−0.75 [−0.98, −0.51]***	−0.75 [−0.98, −0.51]***	−0.60 [−0.84, −0.35]***	−0.64 [−0.87, −0.40]***
55 a 64	−1.78 [−2.03, −1.52]***	−1.78 [−2,03–1.52]***	−1.68 [−1.96, −1.41]***	−1.68 [−1.94, −1.42]***
65 a 74	−3.70 [−4.04, −3.36]***	−3.70 [−4.02, −3.36]***	−3.63 [−3.99, −3.26]***	−3.65 [−3.99, −3.31]***
Female sex	2.07 [1.90,2.25]***	2.07 [1.90, 2.25]***	2.08 [1.87, 2.28]***	2.00 [1.81, 2.20]***
Education				
Incomplete fundamental	1	1	1	1
Complete fundamental	2.23[1.71,2.75]***	2.23[1.71,2.75]***	2.27[1.75,2.80]***	2.20[1.68,2.71]***
High schoool	4.30[3.88,4.72]***	4.30[3.88,4.72]***	4.22[3.79,4.66]***	4.22[3.80,4.65]***
University degree	7.21[6.80,7.63]***	7.22[6.80,7.63]***	7.09[6.66,7.52]***	7.06[6.65,7.47]***
Smoking				
No			1	1
Former			−0.46 [−0.67, −0.25]***	−0.45 [−0.65, −0.24]***
Current			−0.90 [−1.19, −0.62]***	−0.88 [−1.16, −0.61]***
Alcoho l (g/week)				
1–175			1	1
176–350			0.68[0.31, 1.06]***	0.66[0.29, 1.03]**
>350			0.61[−0.03, 1.19] *	0.42[−0.15, 0.98]
Total Col-HDL-C			−0.13[−0.22, −0.04]**	−0.15 [−0.24, −0.06]***

**Table 3:** Final models of multivariate regression with phonemic verbal fluency test (letter F) and diabetes in ELSA-Brasil (baseline 2008–2010) n = 14,444. *Note*. β[CI95%] – coefficient β and 95% confidence interval; R^2^-R^2^ adjusted. Model 1- Adjustment by age, sex and education. Model 2 - Model 1 plus hypertension, coronary disease and depression. Model 3 - Model 2 plus physical activity, smoking, alcohol use, total cholesterol/HDL-C. *p < 0.05; **p < 0.01; ***p < 0.001 Final models included variables with p < 0.05.

Variable	Phonemic verbal fluency (letter F)			
	Model 1 β[CI 95%] R^2^0.17	Model 2 β[CI 95%] R^2^0.18	Model 3 β[CI 95%] R^2^0.18	Final Model β[CI 95%] R^2^0.18
Diabetes	−0.57[−0.75, −0.38]***	−0.52[−0.71, −0.33]***	−0.53[−0.72, −0.33]***	−0.50[−0.69, −0.31]***
Age (years)				
35 a 44	1	1	1	1
45 a 54	−0.33[−0.51, −0.15]***	−0.30[−0.49, −0.12]***	−0.38[−0.57, −0.20]***	−0.37[−0.56, −0.19]***
55 a 64	−0.81[−1.00, −0.61]***	−0.71[−0.91, −0.50]***	−0.83[−1,04–0.63]***	−0.85[−1.05, −0.65]***
65 a 74	−1.36[−1.62, −1.10]***	−1.26[−1.53, −0.99]***	−1.38[−1.66, −1.11]***	−1.37[−1.63, −1.10]***
Female sex	0.13 [−0.01,0.27]	0.11 [−0.03, 0.25]	0.18 [0.03, 0.33]*	0.14 [0.01, 0.28]*
Education				
Incomplete fundamental	1	1	1	1
Complete fundamental	1.58[1.20,1.96]***	1.52[1.13,1.90]***	1.50[1.11,1.89]***	1.57[1.19,1.95]***
High school	3.57[3.26,3.88]***	3.56[3.25,3.87]***	3.52[3.21,3.84]***	3.54[3.23,3.85]***
University degree	5.83[5.53,6.13]***	5.80[5.49,6.10]***	5.76[5.45,6.07]***	5.77[5.46,6.07]***
Hipertension				
No		1	1	1
Yes		−0.35[−0.50, −0.20]***	−0.34[−0.50, −0.19]***	−0.33[−0.48, −0.18]***
Depression				
No		1	1	1
Yes		0.66[0.36,0.96]***	0.66[0.35,0.96]***	0.66[0.37,0.96]***
Smoking				
No			1	1
Former			0.39[0.23,0.55]**	0.40[0.25,0.56]**
Current			0.14[−0.08,0.36]	0.14[−0.07,0.35]
Physical activity				
Low			1	1
Moderate			0.25[0.06,0.44]**	0.27[0.09,0.46]**
High			0.21[−0.07,0.48]	0.17[−0.10,0.44]

**Table 4:** Final models of multivariate regression with semantic phonemic test (animals) and diabetes in ELSA-Brasil (baseline 2008–2010) n = 14,444. *Note*. β[CI95%] – coefficient β and 95% confidence interval; R^2^-R^2^ adjusted. Model 1- Adjustment by age, sex and education. Model 2 - Model 1 plus hypertension, coronary disease and depression. Model 3 - Model 2 plus physical activity, smoking, alcohol use, total cholesterol/HDL-C. *p < 0.05; **p < 0.01; ***p < 0.001 Final models included variables with p < 0.05.

Variable	Semantic phonemic test (animals)			
	Model 1 β[CI 95%] R^2^0.22	Model 2 β[CI 95%] R^2^0.21	Model 3 β[CI 95%] R^2^0.21	Final Model β[CI 95%] R^2^0.21
Diabetes	−0.35[−0.56, −0.14]**	−0.27[−0.50, −0.33]*	−0.30[−0.53, −0.06]*	−0.27[−0.50, −0.04]*
Age (years)				
35 a 44	1	1	1	1
45 a 54	−0.73[−0.94, −0.53]***	−0.72[−0.94, −0.50]***	−0.77[−0.99, −0.55]***	−0.75[−0.96, −0.53]***
55 a 64	−1.61[−1.83, −1.39]***	−1.54[−1.78, −1.30]***	−1.60[−1,84–1.35]***	−1.56[−1.80, −1.32]***
65 a 74	−2.32[−2.62, −2.03]***	−2.21[−2.55, −1.88]***	−2.28[−2.61, −1.94]***	−2.21[−2.54, −1.89]***
Female sex	0.06 [0.10,0.21]	0.12 [−0.05, 0.28]	0.22 [0.04, 0.41]**	0.17 [0.01, 0.34]*
Education				
Incomplete fundamental	1	1	1	1
Complete fundamental	1.36[0.93,1.79]***	1.27[0.78,1.75]***	1.28[0.79,1.76]***	1.30[0.83,1.76]***
High school	3.45[3.10,3.80]***	3.35[2.97,3.75]***	3.37[2.98,3.77]***	3.37[2.99,3.75]***
University degree	6.88[6.53,7.22]***	6.75[6.37,7.13]***	6.77[6.38,7.15]***	6.77[6.39,7.14]***
Hipertension				
No		1	1	1
Yes		−2.82[−0.47, −0.10]**	−0.30[−0.49, −0.11]**	−0.28[−0.46, −0.10]**
Depression				
No		1	1	1
Yes		0.53[0.13,0.93]**	0.57[0.17,0.97]**	0.51[0.13,0.90]**
Smoking				
No			1	1
Former			0.31[0.12,0.50]**	0.33[0.15,0.52]**
Current			−0.01[−0.27,0.26]	0.01[−0.25,0.26]

Therefore, diabetes was associated with a decreased cognitive performance in the memory tests (learning, recall and word recognition tests) (β −0.53 [−10.77; −0.29] adjusted R^2^ 0.21). The verbal fluency test (letter F) performance was decreased among patients with diabetes (β −0.50 [−0.69; −0.31] adjusted R^2^ 0.18) and semantic phonemic test (animals) also was decreased among patients with diabetes (β −0.27[−0.50; −0,04] adjusted R^2^ 0.21), although the magnitude of the association decreased slightly after all adjustments. Finally, an association was found between diabetes and the Trail Making Test B (e^β^1.03 [1.02; 1.06] adjusted R^2^ 0.30), after adjustments (Table 5). Therefore, in the final model, decreased cognitive performance was demonstrated in men, older individuals, patients with low education levels, hypertensive individuals and those who smoke.

**Table 5:** Final models of multivariate regression with log trail-making test B and diabetes in ELSA-Brasil (baseline 2008–2010) n = 14,444. *Note*. e^β^ – β exponential; [CI95%] – confidence interval 95%. Model 1- Adjustment by age, sex and education. Model 2 - Model 1 plus hypertension, coronary disease and depression. Model 3 - Model 2 plus physical activity, smoking, alcohol use, total cholesterol / HDL-C. *p < 0.05; **p < 0.01; ***p < 0.001 Final models included variables with p < 0.05.

Variable	Natural Log of the trail-making test B			
	Model 1 e^β^ [CI 95%] R_2_ 0.29	Model 2 e^β^ [CI 95%] R_2_ 0.29	Model 3 e^β^ [CI 95%] R_2_ 0.30	Final Model e^β^ [CI 95%] R_2_ 0.30
Diabetes	1.05[1.03,1.07]***	1.03[1.01,1.05]**	1.03[1.01,1.05]***	1.03[1.02,1.06]***
Age (years)				
35 a 44	1	1	1	1
45 a 54	1.16[1.14,1.18]***	1.15[1.14,1.18]***	1.16[1.14,1.18]***	1.16[1.14,1.18]***
55 a 64	1.28[1.26,1.31]***	1.27[1.24,1.29]***	1.28[1.25,1.30]***	1.28[1.26,1.31]***
65 a 74	1.42[1.38,1.45]***	1.38[1.34,1.42]***	1.39[1.35,1.43]***	1.40[1.37,1.44]***
Female sex	1.03[1.02,1.04]***	1.03[1.02,1.04]***	1.02[1.01,1.04]***	1.03[1.02,1.04]***
Education				
Incomplete fundamental	1	1	1	1
Complete fundamental	0.87[0.83,0.92]***	0.87[0.82,0.91]***	0.87[0.83,0.92]***	0.88[0.83,0.92]***
High school	0.68[0.65,0.70]***	0.67[0.64,0.70]***	0.67[0.64,0.70]***	0.68[0.65,0.70]***
University degree	0.49[0.47,0.51]***	0.49[0.47,0.51]***	0.49[0.46,0.51]***	0.49[0.47,0.51]***
Hipertension				
No		1	1	1
Yes		1.05[1.03,1.06]***	1.04[1.03,1.06]***	1.05[1.03,1.06]***
Smoking				
No			1	1
Former			0.96[0.95,0.98]***	0.96[0.95,0.98] ***
Current			0.98[−0.96,1.00]	0.98[0.96,1.00]

## Discussion

At baseline in this large Brazilian cohort with relatively young participants (52.0 ± 9.1 years old), an association between cognitive performance in the domains of memory (learning, recall and word recognition tests), phonemic verbal fluency tests (letter F and animals), trail making and diabetes has been shown. This association occurred independently of education and other traditional risk factors (such as lipid levels), comorbidities (such as hypertension), and health-related behaviours (such as smoking).

Previous cross-sectional and longitudinal studies have focused on the substantial epidemiological evidence suggesting that diabetes is associated with cognitive impairment^5,7^. What is unclear is whether there is a specific pattern for impaired function in terms of the affected cognitive domains^26^. Many researchers have described an association between diabetes and cognitive performance in studies in Asia, North America and Europe^2,10,25,27,28^. However, the majority of studies in developed countries have been conducted in cohorts of patients over 60 years old and focused on cognitive diagnoses of conditions such as mild cognitive impairment and dementia^2,4,10,28^.

The influence of diabetes on cognitive performance among young and middle-aged adults, such as the participants in the ELSA-Brasil cohort at baseline, is not well understood, especially in middle- and low-income countries. Our findings show associations between diabetes and cognitive performance in Brazilian individuals, where demographic and nutritional changes are increasing the prevalence of metabolic risk factors and threatening to accelerate the incidence of diabetes and neurodegenerative diseases^29^.

At the present study, diabetes had a significant impact on the performance of memory, phonemic verbal fluency and Trail B tests. In a prospective Dutch study, cognitive performance was measured twice over a five-year interval in 2,613 individuals aged 43–70 years at baseline (1995–2002). They evaluated changes in cognitive performance of individuals with previous or incident diabetes and compared global cognitive performance and specific domains of cognitive function (memory, speed, and cognitive flexibility) among individuals with and without type 2 diabetes. The results showed a decline in global cognitive function in individuals with diabetes that was 2.6 times greater than that in subjects without the disease. Interestingly, the magnitude of cognitive decline in most of the different domains was intermediate in subjects with incident diabetes in comparison with individuals without diabetes or individuals with diabetes that was prevalent at the study baseline. The results of this study seem to indicate that diabetes affects different domains of cognitive functioning at different stages of the disease process^30^.

Previous analysis of this Brazilian cohort has found that education plays a greater role than age in performance on cognitive tests^31^. High education levels were the strongest predictor of maintained cognitive function according to different authors^32,33^. In this study, the impact of education on cognitive performance remained higher than the impact of diabetes, age, and other characteristics.

In the final model relating diabetes and memory tests (learning, recall and word recognition tests), phonemic verbal fluency tests (letter F and animals) and the Trail Making Test B, women presented with better cognitive performance than men, some resemblance to the results found in a German study with middle-aged participants^34^. We found an association between diabetes and cognitive performance, mainly in the domains of memory, attention, concentration, psychomotor speed and executive function. A meta-analysis examining the nature and magnitude of cognitive deficits in individuals with type 2 diabetes was performed to determine the magnitude of the Cohen effect (d) on the cognitive dysfunction of individuals with or without diabetes. The Cohen effect is characterised by differences in the standardised means between the experimental and comparison groups, divided by the standard deviation. The effect size “d” is characterised as small (0.2–0.3), medium (0.4–0.7) and large (≥0.8)^35^. In the meta-analysis, which included 24 studies, a total of 26,137 patients (n = 3,351 with diabetes) met the inclusion criteria. The domains studied were verbal memory (15 studies, n = 4,608, d = −0,28), visual memory (6 studies, n = 1,754, d = −0,26), attention and concentration (14 studies, n = 23,143; d = −0,19), processing speed (16 studies, n = 3,076, d = −0.33), executive function (12 studies, n = 1,784, d = −0.33) and motor function (3 studies, n = 2,374, d = −0.36). The following tests showed the most noticeable performance decreases in samples from patients with diabetes: Rey Auditory Verbal Learning Test (immediate) (d = −0.40), Trail Making Test B (d = −0.39) and the Stroop Part I (d = −0.28)^36^.

To date, insulin has been considered to have neuromodulatory effects that promote the plasticity of synapses^37^. The impairment of insulin signalling, the presence of chronic inflammation and hyperglycaemia, the accumulation of advanced glycation end-products (AGEs) and increases in oxidative stress play an essential role in the pathogenesis of both diabetes and Alzheimer’s disease^38,39^. Inflammatory cytokines produced by macrophages and adipocytes may cross the blood-brain barrier and activate stress kinases, inducing insulin resistance. Moreover, amyloid β protein oligomers can indirectly contribute to brain insulin resistance through the microglial induction of kinases. Neuronal insulin resistance impairs synaptic function and contributes to the neurodegenerative process^40^. In a sample of 998 non-diabetic participants from a subanalysis of the ELSA-Brasil study, an association between a cluster of selected inflammatory biomarkers and poor cognitive scores was also demonstrated in middle-aged women^41^. Micro- and macrovascular disease and unstable metabolic control in individuals with diabetes, including severe hypoglycaemic events, are also critical for cognitive changes ^1,12^.

Furthermore, the loss of brain volume has been described in patients with diabetes, particularly in regions of the hippocampus, thalamus, and cerebellar area^42^. Meta-analysis identified reduced resting-state brain activity in all brain regions in individuals with type 2 diabetes^43^. Although cognitive dysfunction is still not considered to be one of the main complications of diabetes, according to the current recommendations for good practice in the care of individuals with diabetes, it has become important to perform cognitive screening or cognitive assessment of these individuals^13,44,45^.

The strengths of this study include the large sample, methodological rigor in data collection, centralised analysis of the laboratory tests and quality assurance control. The use of this brief battery of neuropsychological tests was standardised and validated for the Brazilian population, thus increasing the reliability of the results^17,46^. In addition, a large set of covariables was evaluated, which allowed adjustment for a wide range of possible confounding factors. However, this study has some limitations. By its cross-sectional design, one cannot infer any causal relationship between cognitive performance and the associated variables. The investigation of the same variables in the follow-up of this cohort may add additional enlightening information. The ELSA-Brasil cohort has a much higher percentage of people with high education levels than the Brazil population. However, it is comparable to the cohorts of other international studies^47^. Moreover, we were unable to evaluate all cognitive tests for each domain, as is the case with large longitudinal studies of adult health in the world. Unfortunately, this comprehensive assessment is logistically impossible in the vast majority of cases.

Although Brazil has a distinct epidemiological profile, the observed associations between diabetes, cognitive performance and metabolic risk factors are similar to those observed in cohorts in developed countries^48,49^. Preventive strategies may be more effective to avoid the worsening of cognition functions in these high-risk individuals^50^. Our data demonstrate that even a brief cognition evaluation is important in assessing the impact of diabetes on the mental health of this population, which may be of great relevance to many similar low- and middle-income countries. The ageing of our population will generate sharp increases in the number of older adults living with diabetes and, possibly, comorbid cognitive impairment. It is estimated, therefore, that the impaired treatment adherence and diabetes self-care represent a major challenge for future health systems around the world, particularly for those with fewer resources^50^.

In conclusion, we found a significant association between diabetes and cognitive function test performance in this relatively young and highly educated Brazilian population.

## Data Availability

The datasets generated during the current study are available from the corresponding author upon reasonable request.
